# Changes of Bacterial Communities in Response to Prolonged Hydrodynamic Disturbances in the Eutrophic Water-Sediment Systems

**DOI:** 10.3390/ijerph16203868

**Published:** 2019-10-12

**Authors:** Haomiao Cheng, Ling Cheng, Liang Wang, Tengyi Zhu, Wei Cai, Zulin Hua, Yulin Wang, Wenfen Wang

**Affiliations:** 1 School of Environmental Science and Engineering, Yangzhou University, Yangzhou 225127, China; 15295512510@163.com (L.C.); zhutengyi@163.com (T.Z.); 007058@yzu.edu.cn (W.C.); wangyulin01986@126.com (Y.W.); 2 School of Hydraulic Science and Engineering, Yangzhou University, Yangzhou 225127, China; wangliang@yzu.edu.cn; 3 Key Laboratory of Integrated Regulation and Resource Development on Shallow Lake of Ministry of Education, College of Environment, Hohai University, Nanjing 210098, China; 4 School of Construction Equipment and Municipal Engineering, Jiangsu Vocational Institute of Architectural Technology, Xuzhou 221008, China; hechengda628@126.com

**Keywords:** bacterial community, water disturbance, 16S rRNA, dissolved oxygen, nutrients, resuspension, racetrack-style flume, lake Chaohu, *Exiguobacterium*, *hgcI_clade*

## Abstract

The effects of hydrodynamic disturbances on the bacterial communities in eutrophic aquatic environments remain poorly understood, despite their importance to ecological evaluation and remediation. This study investigated the evolution of bacterial communities in the water–sediment systems under the influence of three typical velocity conditions with the timescale of 5 weeks. The results demonstrated that higher bacterial diversity and notable differences were detected in sediment compared to water using the 16S rRNA gene sequencing. The phyla *Firmicutes* and *γ-Proteobacteria* survived better in both water and sediment under stronger water disturbances. Their relative abundance peaked at 36.0%, 33.2% in water and 38.0%, 43.6% in sediment, respectively, while the phylum *Actinobacteria* in water had the opposite tendency. Its relative abundance grew rapidly in static control (SC) and peaked at 44.8%, and it almost disappeared in disturbance conditions. These phenomena were caused by the proliferation of genus *Exiguobacterium* (belonging to *Firmicutes*), *Citrobacter*, *Acinetobacter*, *Pseudomonas* (belonging to *γ-Proteobacteria*), and *hgcI_clade* (belonging to *Actinobacteria*). The nonmetric multidimensional scaling (NMDS) and Venn analysis also revealed significantly different evolutionary trend in the three water-sediment systems. It was most likely caused by the changes of geochemical characteristics (dissolved oxygen (DO) and nutrients). This kind of study can provide helpful information for ecological assessment and remediation strategy in eutrophic aquatic environments.

## 1. Introduction

Bacterial communities are responsible for the cycling of nutrients, self-purification, and greenhouse gas emissions in aquatic environments [1,2]. Because of bacteria’s sedentary way of life, their community characteristics in water and sediment are the potential integrative indicators for evaluating the health and stability of aquatic environments, especially for eutrophic ecosystems [3,4]. Therefore, understanding the mechanisms controlling their assembly in water and sediment is vital for water eutrophication treatment and remediation.

The bacterial activities in the water–sediment systems are regulated by past and present environmental conditions [5,6]. Many recent studies have focused on the relationships between bacterial communities and physicochemical properties of the habitats. Generally, higher nutrient levels and temperatures may increase bacterial cell numbers and change community composition [7,8]. Dissolved oxygen (DO) can explain the bacterial community variation in the redundancy analysis targeting freshwater [9]. The pH and salinity are also major elements in shaping bacterial communities [9,10,11].

For the natural aquatic environments, runoff is ubiquitous and changeable, and it is the main physical power to shape the bacterial living environments [12,13]. On one hand, sediment resuspension and interface exchange are aggrandized by flow shearing and turbulence [14,15]. Then, the environmental factors vary significantly, especially the nutrient concentration, DO level, and total suspended solid in the water column. Indirectly, the community composition will undergo differential evolution. On the other hand, high water shear stresses can directly decrease the bacterial diversity, slow down the maturation, and change the community composition [16,17,18,19]. Katharina et al. and Li et al. indicated that hydrodynamic regimes could be considered as a set of reasonable predictors of community composition of benthic biofilms [16,19]. However, the habitats (water and sediment) are interactional and interdependent in the aquatic environments. It is often unclear how the abundance and composition of bacterial communities correlate with water disturbances in water-sediment systems.

Our work aimed to investigate the effects of hydraulic forces on bacterial communities in eutrophic water-sediment systems. Three typical hydrodynamic conditions were simulated by using racetrack-style flumes. Using the 16S rRNA gene sequencing and various multivariate statistical methods, the variation of bacterial community diversity and structure in the water and sediment for different periods were studied. The relationship between bacterial communities and geochemical characteristics were also discussed.

## 2. Materials and Methods

### 2.1. Sampling Sites and Experimental Materials

**Figure 1:**
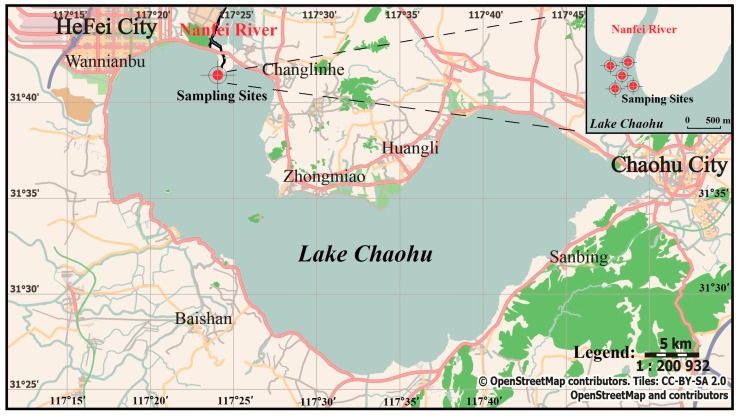
Map of Lake Chaohu in China and the sampling sites (31°41′51″ N, 117°24′16″ E).

Lake Chaohu, located in the lower reaches of the Yangtze River, is one of the five biggest freshwater lakes in China. The sediment of Lake Chaohu has serious eutrophication issues, and it was subject to multiple sources of pollution over the past decades, including industrial sewage, urban runoff, and combined sewer overflows [20,21]. Nanfei River is the largest river flowing into Lake Chaohu and has a large nutrient loading (TN, 5207.5 ton/year; TP, 418.0 ton/year) [22]. The sampling sites were located at the estuary of Nanfei River, which was subject to hydrodynamic regimes perennially. The distribution of the sampling sites is shown in Figure 1, which was drawn by Inkscape 0.92.4 and Openstreetmap (https://www.openstreetmap.org). Due to the main active and homogeneous layer affecting by water disturbances, the surface sediment samples (0–5 cm) were collected by using a Peterson grab sampler (Punsen, Changzhou, China) [23,24]. The details of the physicochemical characteristics of the sediment are listed in Table S1. All the sediment samples in each site were gathered, mixed, and fully homogenized. After putting into sterile polyethylene bags, the samples were immediately transported to the laboratory and kept at −80 °C until the experiment began.

### 2.2. Experimental Facilities and Method

**Figure 2:**
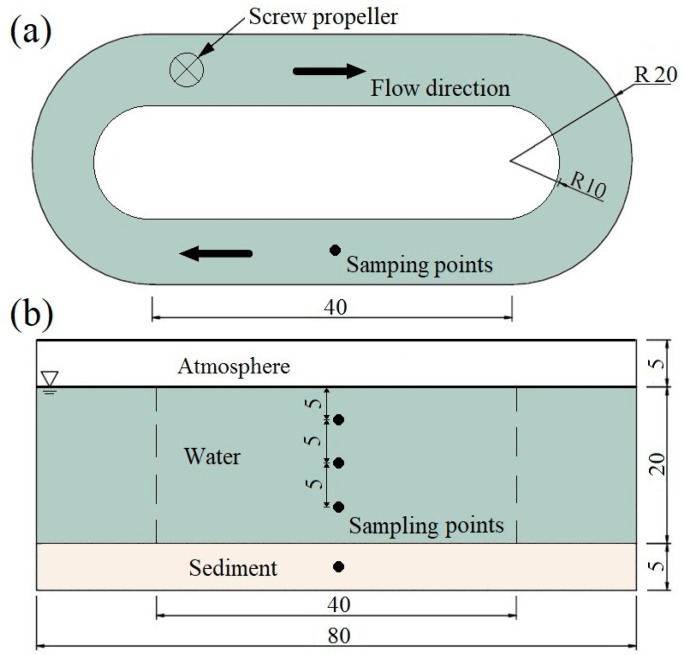
Sketch of the experimental facility (cm) and the sampling points. (**a**) planform; (**b**) side elevation. A screw propeller was used to simulate different velocity conditions by adjusting rotational-speed.

Three parallel racetrack-style flumes were employed to conduct the following water disturbance experiments. Our previous study has proved that this type of flumes was effective in simulating the water–sediment interface exchange under the influence of different stream courses [25]. The size and structure are illustrated in Figure 2. The homogenized sediment samples were divided into three parts and spread evenly in the bed of the flumes to a thickness of 5 cm. The experiment employed ultrapure water as the overlying water to avoid the impact of the complex raw water quality. A 20 cm layer of ultrapure water was slowly injected into the flumes via a siphon without disturbing the sediment. Then these water-sediment systems were left undisturbed for a week to serve as a near natural substratum.

The characteristic of velocity is the most critical factor in the change of hydrodynamic conditions. According to in-situ velocity observation and previous reports in the sampling area, three frequent velocities were carried out simultaneously via adjusting rotational-speed of the screw propeller [20,26]. They were static control (SC), slow velocity condition (SVC), and fast velocity condition (FVC). The corresponding average vertical velocities ($\bar{U}$) in each condition were set as 0 cm/s, 4.5 cm/s, and 20.8 cm/s, respectively. The experiment lasted for five weeks. During the experiment, the water was recirculated in the flumes and the velocity structures in each condition were controlled steadily and continuously.

The sample collection of water and sediment was conducted weekly. The sampling points are illustrated in Figure 2. Approximately 300 mL of water was collected from each point using a layered hydrophore. The parallel water samples from three points were mixed for homogenization. Each mixed sample was filtered through quartz fiber filters (3 μm pore size) to eliminate suspended solids. Bacterial cells were collected onto polyethersulfone membrane filters (0.22 μm pore size) by using a vacuum filter. The filter membranes with bacterial cells were transferred to sterile tubes and stored at −80 °C until the DNA could be extracted [27]. Meanwhile, the filtrates (100 mL) were used to analyze total phosphorus (TP) and total nitrogen (TN). TP and TN were analyzed by the molybdenum blue and Kjeldahl spectrophotometry method, respectively. For the sediment sampling, a cylindrical sampler (5 cm I.D., 50 cm in length) was used to collect sediment samples. After homogenization and careful washing by sterile deionized water, approximately 4 g of sample was placed in the sterile tube hermetically, than stored at −80 °C until the DNA could be extracted [28]. The water depth was kept constant by adding ultrapure water after each sampling.

Moreover, the flow structures in each flume were controlled for stability during the experiment. The velocities were monitored daily by using an acoustic Doppler velocimeter (Nortek, Oslo, Norway) from the bottom to water surface with 1-cm intervals. The DO and pH values were measured using a HACH HQ30d portable meter (HACH Company, Loveland, CO, USA) daily. The experimental temperature was kept stable at 15 ± 0.3 °C.

### 2.3. Sample Analysis

The 16S rRNA gene analysis was applied to determine the bacterial communities in the water and sediment samples. The brief process is as follows: the total DNA from the homogenized water and sediment samples was extracted using the Power Water/Soil Sterivex DNA Isolation Kit (MO BIO Laboratories, Inc., Carlsbad, CA, USA), following the manufacturer’s instructions. DNA quality was checked on a 1% agarose gel by spectrophotometric analysis with a NanoDrop ND-2000 (Thermo Fisher Scientific, Wilmington, CA, USA). Amplification of the target fragment was V4 region in the 16S rRNA gene by the primers F515 (GTGYCAGCMGCCGCGGTAA) and R806R (GGACTACNVGGGTWTCTAAT) [29]. The polymerase chain reaction (PCR) amplification was performed on a BioRad S1000 thermal cycler (Bio-Rad Laboratory, Hercules, CA, USA). The reaction conditions were tripartite: preheating at 94 °C for 5 min; denaturation at 94 °C for 30 s, cycle 31 times; annealing at 52 °C for 30 s; extension at 75 °C for 45 s; lasting 72 °C for 10 min. After PCR amplification, the PCR products were stored at 4 °C and then sent to Guangdong Magigene Biotechnology Co., Ltd. (Guangzhou, China) for Illumina HiSeq sequencing. The quality was checked by the MOTHUR software. Then, the sequences were trimmed and aligned using the SILVA reference database. The sequences were clustered into operational taxonomic units (OTUs) with a 97% similarity identity cutoff. More detailed information of the 16S rRNA gene analysis is described in References [10,30].

### 2.4. Multivariate Statistics

Statistical analyses were conducted using the SPSS 19.0 (IBM, Armonk, NY, USA) and R 3.6.1 (R Development Core Team Vienna, Austria) (https://www.reproject.org/). The rarefaction curves were analyzed using MOTHUR, with an OTU defined at 97% similarity. The bacterial diversity, richness, and relative abundance were analyzed using the R package “vegan” [31,32]. Nonmetric multidimensional scaling (NMDS) and Venn analysis were applied to determine the spatial relationships among bacterial communities. NMDS analysis was calculated based on the Bray-Curtis dissimilarity matrix by using the R package “vegan”. Venn diagrams were generated to visualize OTUs common to SC, SVC, and FVC at week 5 using the Venn tool [33]. It is statistically significant in the t-test if the *p*-value is smaller than 0.05.

## 3. Results and Discussion

### 3.1. Physicochemical Properties in the Water-Sediment Systems

**Figure 3:**
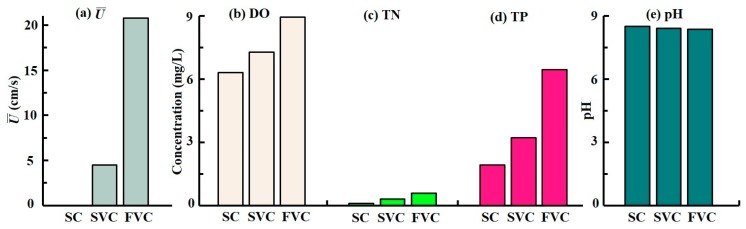
Velocity conditions and physicochemical properties in each water-sediment system. (**a**) Average vertical velocity ($\bar{U}$), (**b**) Dissolved oxygen (DO), (**c**) Total phosphorus (TP), (**d**) Total nitrogen (TN), (**e**) pH. SC: static control, SVC: slow velocity condition, FVC: fast velocity condition. All the data were the average values from 0 weeks to 5 weeks, and were statistically significant with a *p*-value < 0.05.

No discernible differences in the physico-chemical properties were observed in any water-sediment system over time, implying that the bacterial living environment remained stable in each system from week 0 to week 5. This dynamic equilibrium was similar to previous studies [26]. The average values of DO, TN, TP, and pH were analyzed and shown in Figure 3. The DO values increased with the enhanced water disturbances from 6.3 mg/L in the SC to 9.0 mg/L in the FVC. Due to the strong flow turbulence, the oxygen can be fully mixed, oxygen exchange was promoted, and extra oxygen could be held in the overlying water [34,35]. Meanwhile, nutrient concentrations (TN and TP) showed a similar tendency with DO. TN and TP were 0.6 mg/L and 6.5 mg/L in the FVC, which were 5.4 times and 3.3 times that in the SC. This was due to the sediment resuspension, which released large amounts of nutrients from the eutrophic sediment into the overlying water [12,36]. Similar results were observed in previous research [37,38]. The pH showed no significant statistical differences, and the mean value was 8.43 ± 0.09.

### 3.2. Diversity of Bacterial Communities

Sequencing data were generated from the 18 water samples and 18 sediment samples. Table S2 is an overview of the OTU numbers and diversity indexes of these samples. As a result, a total of 721 709 sequences (water: 388 398 and sediment: 333 311) was obtained after quality filtering and trimming. The sequences were assigned to 53 050 OTUs (water: 10 934 and sediment: 42 116) with a 97% sequence identity threshold. Rarefaction curves of water and sediment samples are shown in Figure S1 (Supplementary Material). These rarefaction curves approached to a saturation plateau, which indicated that most of the bacterial OTUs were captured.

In the water, the bacterial diversity and richness in the SVC were the highest (mean OTUs of 687, Chao index of 861, Shannon index of 5.71, and Simpson index of 0.06), followed by SC (mean OTUs of 621, Chao index of 755, Shannon index of 4.78, and Simpson index of 0.11) and FVC (mean OTUs of 607, Chao index of 862, Shannon index of 4.70, and Simpson index of 0.12). There might be two reasons: on the one hand, the bacterial cells from the sediment would release into the water column though the resuspension process [39,40]; on the other hand, strong water disturbance (especially FVC) would have the inhibition effect on bacterial diversity and richness [16,41].

In the sediment, as the water velocity increased, the OTUs showed a decreasing trend (*p* < 0.1), and the mean numbers were 2568 in the SC, 2352 in the SVC, and 2099 in the FVC, respectively. Similar tendencies were found in the Chao richness index and the Shannon and Simpson diversity indexes in the sediment (*p* < 0.1). The results indicated that strong currents inhibited the bacterial diversity and richness, as the flow shearing force and friction exerted on the bacteria induced the loss of bacterial communities [42].

After comparing the OTU numbers of water and sediment (see Table S2), the mean value of OTUs was 607 in water samples (range: 321–848), which was far less than that in sediment samples (mean: 2340, range: 1246–2995). Unsurprisingly, the Chao richness indexes and Shannon and Simpson diversity indexes revealed a lower level of biodiversity in water (mean Chao index of 826, Shannon index of 5.06, Simpson index of 0.099) than that in sediment (mean Chao index of 2499, Shannon index of 7.58, Simpson index of 0.047). This can be explained by the fact that bacterial communities clustered by environment habitats and sediment have higher adaptability to provide more micro niches [10,43].

### 3.3. Composition Evolution of Bacterial Community

#### 3.3.1. Phylum

(1) Water

**Figure 4:**
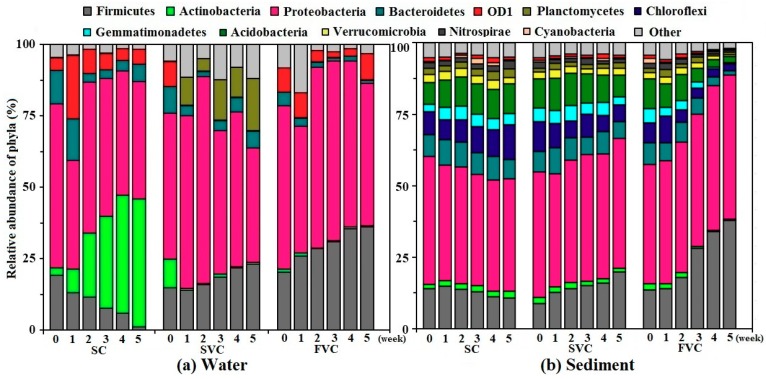
Relative abundance of bacterial reads classified at phylum level along the timescale (weeks 0–5) obtained from Ribosomal Database Project (RDP) classifier analysis (mean relative abundance > 1.0%). (**a**) Water, (**b**) Sediment. SC: static control, SVC: slow velocity condition, FVC: fast velocity condition.

The changes of the bacterial communities in response to water disturbances were investigated. In total, 48 phyla in the water samples were detected by the RDP classifier. As Figure 4a shows, 6 dominant phyla accounted for 94.1% of the total bacterial sequences. They were *Proteobacteria*, *Firmicutes*, *Actinobacteria*, *Bacteroidetes*, *OD*1, and *Planctomycetes*, which included more than 1.0% of the total bacterial sequences. Notably, the most dominant bacterial phylum were *Proteobacteria*, and the mean relative abundance was 52.2%. The relative abundance of *Proteobacteria* in each condition was fluctuant. Among different classes of *Proteobacteria*, *γ*-*Proteobacteria* were predominant (mean: 19.5%) and *γ*-*Proteobacteria* gradually increased with the enhanced water disturbances. The reads of *γ*-*Proteobacteria* in the SC were 19.2% (0 weeks), and then decreased to 1.9% (5 weeks). In contrast, the relative abundance of *γ*-*Proteobacteria* in the FVC increased from 22.3% (0 weeks) to 33.2% (5 weeks), as shown in Figure S2a (Supplementary Material). *Firmicutes* showed a similar tendency with *γ*–*Proteobacteria* (Figure 4a). The reads of *Firmicutes* decreased from 19.1% and 1.2% in the SC and increased from approximate 17.4% to 23.1% (SVC) and 36.0% (FVC), respectively. The increase in the reads of both *γ–Proteobacteria* and *Firmicutes* with water disturbances might be supported by DO and nutrient concentrations (TP and TN) growth. Similar phenomena were reported in previous studies [28,44,45]. Moreover, the reads of *Bacteroidetes* and *OD*1 decreased in the SVC and FVC, between weeks 0 and 5, due to the mass proliferation of *γ*-*Proteobacteria* and *Firmicutes*. Meanwhile, the reads of *Actinobacteria* presented a different trend. The reads of *Actinobacteria* in the SC were 2.7% (0 weeks), and then peaked at 44.8% (5 weeks). The reads of *Actinobacteria* almost disappeared under the influence of water disturbances (around 0.5% between week 1 and 5). This might result from the fact that *Actinobacteria* was negatively correlated with TP levels [28,45]. In the SC, due to the mass proliferation of *Actinobacteria*, the corresponding reads of *Firmicutes*, *Proteobacteria*, and *Bacteroidetes* decreased from 19.1%, 57.3% and 12.0% to 1.2%, 40.9%, and 3.1%, respectively. The fluctuations of the reads of *OD*1 were detected (mean: 8.3%, range: 4.2–22.2%).

(2) Sediment

Relatively richer bacterial communities were detected in sediment samples (shown in Figure 4b). An examination of the database revealed 66 phyla, and there were 12 dominant phyla in sediment (mean relative abundance > 1.0%) during the 5-week period. They accounted for 95.7% of the total bacterial sequences, including *Proteobacteria, Firmicutes, Actinobacteria*, *Bacteroidetes*, *OD*1, *Planctomycetes*, *Chloroflexi*, *Gemmatimonadetes*, *Acidobacteria*, *Verrucomicrobia*, *Nitrospirae* and *Cyanobacteria*. These dominant phyla were basically consistent with the previous reports in the sediment of Lake Chaohu [46,47]. The most dominant bacterial phylum in each condition were also *Proteobacteria*, which constituted a high proportion from 38.7% to 50.6% (mean: 42.7%). The *γ-Proteobacteria* was also the most dominant group, with a mean value of 21.7% among the classes of *Proteobacteria* (shown in Figure S2b, Supplementary Material). In the SC, the relative abundance of each phylum fluctuated within a narrow range (weeks 0–5), and it indicated that the bacterial communities in the sediment of SC remained basically stable. In the SVC and FVC, the relative abundance of *γ-Proteobacteria*, *Firmicutes* and *Actinobacteria* showed a similar regularity to that in water. The reads of *γ-Proteobacteria* and *Firmicutes* increased sharply in the FVC from 16.9%, 13.6% (0 weeks) to 43.6%, 38.0% (5 weeks). Meanwhile, the relative abundance of *Actinobacteria* showed an opposite trend. The reads of *Actinobacteria* in the SVC and FVC decreased from 2.2% to 1.1% and 0.5% (weeks 0–5), respectively. As the mass proliferation of *γ-Proteobacteria* and *Firmicutes* increased, the relative abundance of other 10 phyla decreased gradually.

#### 3.3.2. Genus

(1) Water

**Figure 5:**
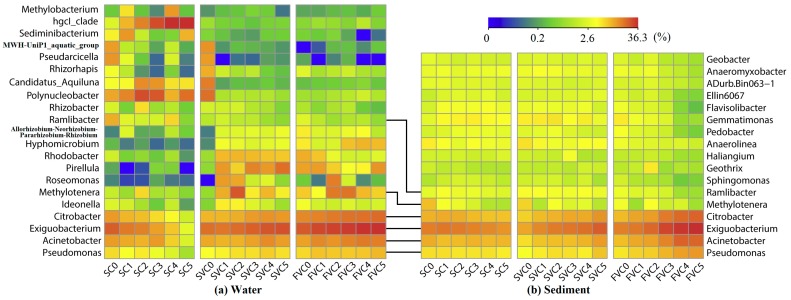
Relative abundance of bacterial reads classified at genus level along the timescale (week 0–5) obtained from Ribosomal Database Project (RDP) classifier analysis (mean relative abundance > 0.5%). (**a**) Water, (**b**) Sediment.

At the genus level, there were 879 genera of known bacteria in water samples, and 21 bacterial genera accounted for more than 0.5% of all sequences on average (see Figure 5a). The genus *Exiguobacterium* had the highest relative abundance (mean: 18.3%), belonging to phylum *Firmicutes*. This genus significantly accumulated under the action of strong water flow (*p* < 0.05). Its relative abundance in the SC decreased from 18.4% to 1.1%, and increased from approximately 16.9% to 22.3% in the SVC and 34.7% in the FVC, respectively. Previous research reported that *Exiguobacterium* was a genus potentially sensitive to eutrophic water (especially nitrate) [48,49]. The proliferation of *Exiguobacterium* under the disturbance conditions might result from the increased TN concentrations (Figure 3). A similar tendency was found in genera *Citrobacter* (mean: 7.9%), *Acinetobacter* (mean: 7.5%), and *Pseudomonas* (mean: 2.9%). In the FVC, their relative abundance gradually increased from 10.1%, 7.0%, and 3.4% to 15.1%, 11.9%, and 5.2%, respectively. These genera are aerobic and nutrient-loving, belonging to *γ-Proteobacteria* [50,51]. The higher DO and nutrient concentrations, which induced by water disturbance, resulted in the growth of these genera. In addition, a significantly higher abundance of genus *hgcI_clade* of the phylum *Actinobacteria* was found in the SC, which peaked at 41.7% (5 week). By contrast, the relative abundance of *hgcI_clade* in the SVC and FVC were very low (range: 0.1–0.4%). Regarding to the previous works, the genus *hgcI_clade* is known to have competitive advantage over others in an oligotrophic freshwater [52,53]. In this experiment, nutrients were limited in the overlying water during periods of no flow (SC) compared to SVC and FVC. In this case, *hgcI_clade* was hard to survive and grow in the SVC and FVC due to their high nutrient concentrations. Other genera had no regular variations in each condition.

(2) Sediment

Among the 1121 bacterial genera detected in sediment samples, the 17 dominant bacterial genera (mean relative abundance > 0.5%) are shown in Figure 5b. The top 6 genera were shared in both sediment and water in each condition, including *Exiguobacterium* (mean: 15.4%), *Citrobacter* (mean: 7.4%), *Acinetobacter* (mean: 7.0%), *Pseudomonas* (mean: 3.7%), *Methylotenera* (mean: 1.8%), and *Ramlibacter* (mean: 1.5%), as the genera would exchange between the interactional and interdependent habitats (water and sediment) [54]. Similarly to the water, the abundances of genera *Exiguobacterium*, *Citrobacter*, *Acinetobacter*, and *Pseudomonas* showed a significant increase with the enhanced water disturbances (*p* < 0.05). In the FVC, their relative abundance increased from 11.6% to 36.3%, 5.1–17.7%, 4.8–16.7%, and 2.7–8.0%, respectively. The physicochemical properties of the sediment were basically stable along the timescale from week 0 to week 5 (see Table S1). Thus, the regular change of the bacteria in the sediment might be caused by hydrodynamic shear stress [54]. Moreover, no significant regularity was found for the abundance of other genera.

### 3.4. Spatial Distribution of Bacterial Communities

**Figure 6:**
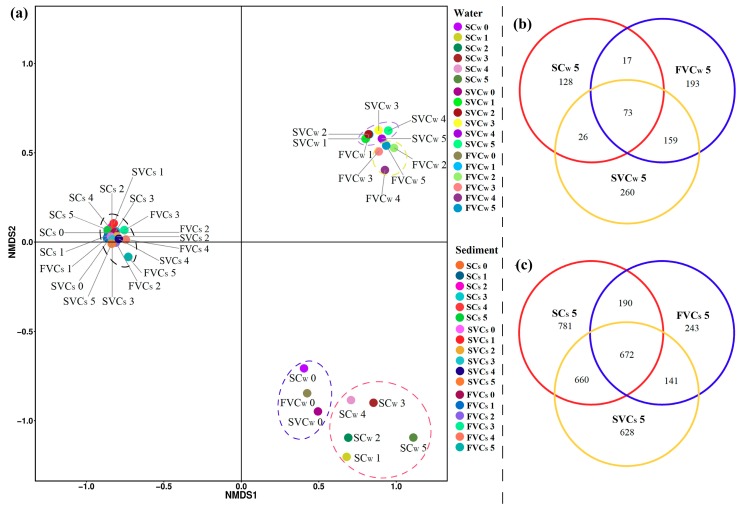
(**a**) Non-metric multidimensional scaling (NMDS) ordination visualization of the bacterial communities in the water and sediment along the timescale (week 0–5). (**b**) Venn diagram of water samples at 5 weeks. (**c**) Venn diagram of sediment samples at 5 weeks. The detailing taxon shared were shown in Table S3. SC: static control, SVC: slow velocity condition, FVC: fast velocity condition. The subscript *S* denotes the sediment and *W* denotes the water.

The NMDS analysis was conducted to determine the UniFrac distance of the bacterial community data in water and sediment samples. It can reveal the dynamics and distribution patterns of community compositions at the OTU-level taxa. The NMDS plots of water and sediment samples are shown in Figure 6a. There was a considerable separation in water samples between no flow (SC_W_) and disturbance conditions (SVC_W_ and FVC_W_). It indicated that the microorganisms in overlying water could resettle and vary in the hydrodynamic and resuspension processes. A relatively clear separation was observed between SVC_W_ and FVC_W_. This may be due to the combined contributions of different physicochemical properties. The bacterial community compositions in sediment (SC_S_, SVC_S,_ and FVC_S_) were distributed in different positions from water samples, which could be related to different habitats. Meanwhile, all SC_S_, SVC_S,_ and FVC_S_ were clustered. This phenomenon indicated that interfacial shear stress had limited contribution to the *β*-diversity in the sediments of each system.

Venn diagrams can be used to better understand the proportion of shared and unique OTUs generated at each platform. The OTUs comparison of water and sediment samples at week 5 are displayed in Figure 6b,c. In the water, there were 856 OTUs in the three samples altogether, with 128 unique in the SC_W_, 260 in the SVC_W,_ and 193 in the FVC_W_ (Figure 6b). Compared to the SC_W_, the SVC_W_ and FVC_W_ were found to share a large number of OTUs (159 OTUs) in common, and it indicated that water disturbances significantly changed the bacterial communities in water. As shown in Figure 6c, there were 3315 in the three sediment samples altogether. The unique OTUs of SC occupied the largest proportion (23.6%) of the total, followed by SVC_S_ (18.9%) and FVC_S_ (7.3%). The result also indicated that stronger currents had the inhibiting effect on bacterial communities of sediment. The details of the shared taxon among groups are shown in Table S3.

## 4. Conclusions

This study provides insights into the bacterial communities influenced by hydrodynamic disturbances in eutrophic water–sediment systems. The bacterial communities in sediment had a higher diversity than the water. The strong water disturbances had the inhibiting effect on bacterial communities in both water and sediment. However, the relative abundance of phyla *Firmicutes* and *γ–Proteobacteria* were found to increase regularly with the enhanced water disturbances, due to the proliferation of genera *Exiguobacterium* (peaked at 34.7% for water and 36.3% for sediment), *Citrobacter* (peaked at 15.1% for water and 17.7% for sediment), *Acinetobacter* (peaked at 11.9% for water and 16.7% for sediment) and *Pseudomonas* (peaked at 5.2% for water and 8.0% for sediment). The genus *hgcI_clade* (belonging to phylum *Actinobacteria*) had a high abundance in the static system (peaked at 41.7% in the water), while it was hard to survive in both water and sediment under the water disturbances. The regular changes of these genera were most likely caused by the changes of DO and nutrients in overlying water, when hydrodynamic and resuspension processes were induced. The results give a better understanding of the responses of bacterial communities to hydrodynamic disturbances in aquatic environments.

## Supplementary Materials

The following are available online at https://www.mdpi.com/1660-4601/16/20/3868/s1, Table S1: Physical and chemical properties of experimental sediment, Table S2: Comparison of OTU numbers and diversity indexes of bacterial community in the water-sediment systems over time, Table S3: The detailing taxon shared among groups of water and sediment samples at week 5, Figure S1: Rarefaction curves of OTUs richness clustered at 97% sequence identity. (a) Water, (b) Sediment. SC: static control, SVC slow velocity condition, FVC: fast velocity condition. The subscript *S* denotes the sediment and *W* denotes the water, Figure S2: Relative abundance of *γ-Proteobacteria* along the timescale (week 0–5). (a) Water, (b) Sediment. SC: static control, SVC slow velocity condition, FVC: fast velocity condition.
