# Supplementary material for: Changes of Bacterial Communities in Response to Prolonged Hydrodynamic Disturbances in the Eutrophic Water-Sediment Systems
Source: Int J Environ Res Public Health. 2019 Oct 12;16(20):3868. doi: 10.3390/ijerph16203868 (PMC6843157; doi:10.3390/ijerph16203868)
Supplement: Supplementary file 1 [file ijerph-16-03868-s001.pdf]

International Journal of
Environmental Research and Public Health

MDPI

Supplementary Material: Changes of Bacterial Communities in Response to Prolonged Hydrodynamic Disturbances in the Eutrophic Water-Sediment Systems

Table S1. Physical and chemical properties of experimental sediment.

pH	Particle Size Distribution (%)			TOC	D_{50}	TP	TN	TBBPA
	Clay	Silt	Sand	(%)	(μm)	(mg/kg)	(mg/kg)	(μg/kg)
**7.94**	**10.12**	**73.45**	**16.43**	**1.51**	17.32	836.1	1654.3	632.7

Clay: <3.9 μm; Silt: 3.9–63 μm; Sand: 63–2000 μm. TOC: Total organic carbon content; D_{50}: Media diameter; TP: Total phosphorus; TN: Total nitrogen; TBBPA: TBBPA concentration. TOC was measured by a Liqui TOC II Analyzer. Particle size distribution and D_{50} were measured by the Malvern Mastersizer 2000 (Malvern Instruments Ltd., Malvern, British). The data are the average value analyzed from additional sediment samples. All the values are significant, $p < 0.05$. The experimental sediment was the same as those in our previous study [1,2].

Table S2. Comparison of OTU numbers and diversity indexes of bacterial community in the water-sediment systems over time.

Sample	Time	Reads [a]	OTU [b]	Chao	Shannon	Simpson
			**Water**			
SCw 0	Week 0	23,892	782	902.6	5.11	0.069
SCw 1	Week 1	30,264	760	744.9	5.35	0.053
SCw 2	Week 2	22,588	611	716.4	4.64	0.091
SCw 3	Week 3	27,229	585	667.7	4.09	0.120
SCw 4	Week 4	20,707	578	733.6	4.60	0.115
SCw 5	Week 5	23,860	413	762.2	4.90	0.241
SVCw 0	Week 0	29,174	764	1026.9	6.47	0.075
SVCw 1	Week 1	26,407	848	909.4	5.40	0.037
SVCw 2	Week 2	28,135	574	929.5	6.28	0.066
SVCw 3	Week 3	21,818	739	806.5	5.62	0.050
SVCw 4	Week 4	27,940	684	730.0	5.59	0.068
SVCw 5	Week 5	25,936	518	762.2	4.90	0.071
FVCw 0	Week 0	28,166	624	874.9	5.95	0.061
FVCw 1	Week 1	30,154	788	931.7	5.31	0.090
FVCw 2	Week 2	25,405	352	735.0	4.32	0.122
FVCw 3	Week 3	27,985	532	895.3	4.40	0.133
FVCw 4	Week 4	20,226	321	998.8	4.07	0.162
FVCw 5	Week 5	27,122	461	740.2	4.13	0.164
			**Sediment**			
SCs 0	Week 0	42,631	2995	2681.5	8.07	0.026
SCs 1	Week 1	23,720	2101	2535.1	8.03	0.027
SCs 2	Week 2	24,254	2253	2736.2	8.19	0.024
SCs 3	Week 3	31,994	2607	2579.9	8.24	0.021
SCs 4	Week 4	33,475	2826	2783.8	8.53	0.018
SCs 5	Week 5	30,958	2628	2580.3	8.54	0.015
SVCs 0	Week 0	24,709	2335	2628.4	8.67	0.011
SVCs 1	Week 1	36,449	2783	2684.0	8.38	0.018
SVCs 2	Week 2	29,739	2334	2527.7	8.04	0.025
SVCs 3	Week 3	29,580	2277	2561.1	7.94	0.028
SVCs 4	Week 4	26,398	2173	2460.3	7.76	0.031
SVCs 5	Week 5	25,540	2210	2425.1	7.34	0.051

FVCs 0	Week 0	29,538	2471	2662.6	8.16	0.022
FVCs 1	Week 1	40,518	2780	2642.4	8.03	0.029
FVCs 2	Week 2	23,551	2149	2502.8	7.55	0.040
FVCs 3	Week 3	23,424	2042	2374.6	6.20	0.099
FVCs 4	Week 4	31,328	1906	1898.7	4.62	0.163
FVCs 5	Week 5	19,610	1246	1714.7	4.06	0.196

[a] Reads after filtering, trimming and normalizing. [b] The operational taxonomic units (OTUs) were defined with 97% similarity. SC: static control, SVC slow velocity condition, FVC: fast velocity condition. The subscript *S* denotes the sediment and *W* denotes the water.

Table S3. The detailing taxon shared among groups of water and sediment samples at week 5.

**Taxa**	**OTUs in Sediment**			**Taxa**	**OTUs in Water**		
	**SCs**	**SVCs**	**FVCs**		**SCw**	**SVCw**	**FVCw**
Acidobacteria	226	196	116	Acidobacteria	4	25	21
Actinobacteria	94	67	39	Actinobacteria	13	26	24
Armatimonadetes	29	23	4	Armatimonadetes	1	3	7
BRC1	6	4	3	/	/	/	/
Bacteria	36	25	22	Bacteria	4	0	2
Bacteroidetes	187	190	122	Bacteroidetes	22	61	39
Caldiserica	2	2	1	Caldiserica	0	0	1
Calditrichaeota	3	3	1	/	/	/	/
Chlamydiae	9	10	5	Chlamydiae	16	6	6
Chloroflexi	303	201	144	Chloroflexi	1	10	17
Cyanobacteria	24	26	13	Cyanobacteria	1	33	24
Dadabacteria	1	1	0	/	/	/	/
Deinococcus-Thermus	4	2	1	Deinococcus-Thermus	1	2	2
Dependentiae	4	4	1	Dependentiae	2	0	1
Edwardsbacteria	1	0	0	/	/	/	/
Elusimicrobia	13	18	8	/	/	/	/
Epsilonbacteraeota	1	2	1	Epsilonbacteraeota	0	1	0
FBP	0	1	0	/	/	/	/
Fibrobacteres	4	5	0	/	/	/	/
Firmicutes	74	64	48	Firmicutes	4	9	15
Gemmatimonadetes	81	78	35	Gemmatimonadetes	5	13	9
Hydrogenedentes	5	4	3	/	/	/	/
Kiritimatiellaeota	11	17	5	/	/	/	/
Latescibacteria	25	23	13	Latescibacteria	1	0	1
Lentisphaerae	4	6	2	/	/	/	/
Modulibacteria	1	0	0	/	/	/	/
Nitrospinae	5	3	3	Nitrospinae	0	2	1
Nitrospirae	42	38	32	Nitrospirae	2	2	5
Omnitrophicaeota	21	18	18	Omnitrophicaeota	1	0	1
PAUC34f	0	1	0	/	/	/	/
Patescibacteria	88	111	52	Patescibacteria	36	7	16
Planctomycetes	157	114	75	Planctomycetes	3	56	40
Proteobacteria	699	705	389	Proteobacteria	124	239	190
Rokubacteria	9	6	5	/	/	/	/
Spirochaetes	37	34	25	Spirochaetes	1	0	4
Synergistetes	2	1	0	/	/	/	/
TA06	1	0	2	/	/	/	/
Tenericutes	0	0	1	Tenericutes	0	1	1
Verrucomicrobia	72	78	50	Verrucomicrobia	2	18	14
WOR-1	2	0	0	WOR-1	0	0	1
WPS-2	1	2	1	WPS-2	0	2	0
WS1	6	4	0	/	/	/	/
WS2	2	1	1	/	/	/	/
WS4	3	4	2	/	/	/	/

Zixibacteria	8	9	3	Zixibacteria	0	2	0
Total	2303	2101	1246	Total	244	518	442

The operational taxonomic units (OTUs) were defined with 97% similarity. SC: static control, SVC slow velocity condition, FVC: fast velocity condition. The subscript *S* denotes the sediment and *W* denotes the water.

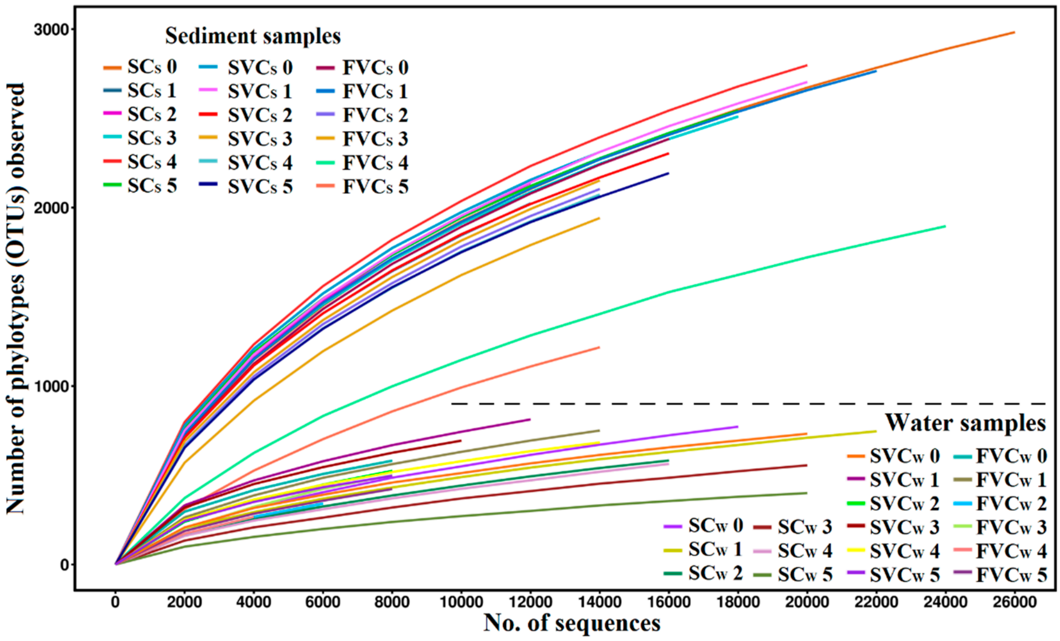

Figure S1. Rarefaction curves of OTUs richness clustered at 97% sequence identity. (**a**) Water, (**b**) Sediment. SC: static control, SVC slow velocity condition, FVC: fast velocity condition. The subscript *S* denotes the sediment and *W* denotes the water.

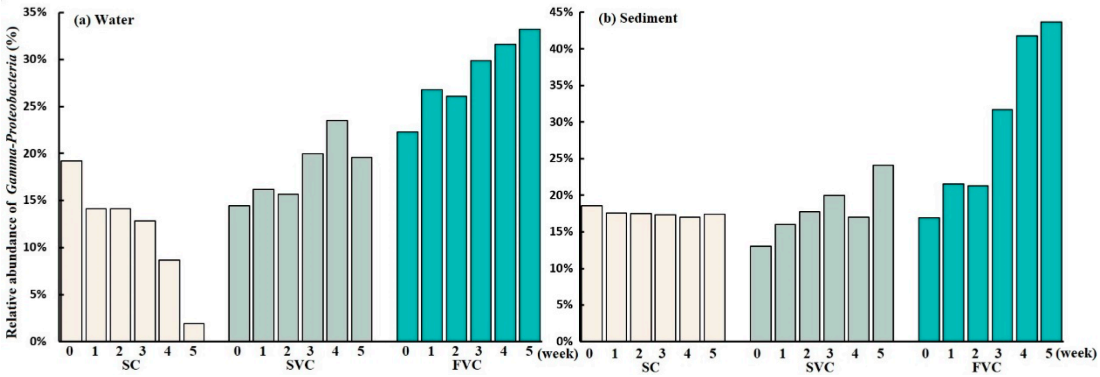

Figure S2. Relative abundance of *γ-Proteobacteria* along the timescale (week 0–5). (**a**) Water, (**b**) Sediment. SC: static control, SVC slow velocity condition, FVC: fast velocity condition.

References

1. Cheng, H.M.; Hua, Z.L. Distribution, release and removal behaviors of tetrabromobisphenol A in water-sediment systems under prolonged hydrodynamic disturbances. *Sci. Total Environ.* **2018**, *636*, 402–410.
2. Cheng, H.M.; Hua, Z.L. Effects of hydrodynamic disturbances and resuspension characteristics on the release of tetrabromobisphenol A from sediment. *Environ. Pollut.* **2016**, *219*, 785–793.

© 2019 by the authors. Submitted for possible open access publication under the terms and conditions of the Creative Commons Attribution (CC BY) license (http://creativecommons.org/licenses/by/4.0/).
